# Disruption of diphthamide synthesis genes and resulting toxin resistance as a robust technology for quantifying and optimizing CRISPR/Cas9-mediated gene editing

**DOI:** 10.1038/s41598-017-15206-x

**Published:** 2017-11-13

**Authors:** Tobias Killian, Steffen Dickopf, Alexander K. Haas, Claudia Kirstenpfad, Klaus Mayer, Ulrich Brinkmann

**Affiliations:** Roche Pharma Research and Early Development (pRED), Therapeutic Modalities - Large Molecule Research, Roche Innovation Center Munich, Nonnenwald 2, D-82372 Penzberg, Germany

## Abstract

We have devised an effective and robust method for the characterization of gene-editing events. The efficacy of editing-mediated mono- and bi-allelic gene inactivation and integration events is quantified based on colony counts. The combination of diphtheria toxin (DT) and puromycin (PM) selection enables analyses of 10,000–100,000 individual cells, assessing hundreds of clones with inactivated genes per experiment. Mono- and bi-allelic gene inactivation is differentiated by DT resistance, which occurs only upon bi-allelic inactivation. PM resistance indicates integration. The robustness and generalizability of the method were demonstrated by quantifying the frequency of gene inactivation and cassette integration under different editing approaches: CRISPR/Cas9-mediated complete inactivation was ~30–50-fold more frequent than cassette integration. Mono-allelic inactivation without integration occurred >100-fold more frequently than integration. Assessment of gRNA length confirmed 20mers to be most effective length for inactivation, while 16–18mers provided the highest overall integration efficacy. The overall efficacy was ~2-fold higher for CRISPR/Cas9 than for zinc-finger nuclease and was significantly increased upon modulation of non-homologous end joining or homology-directed repair. The frequencies and ratios of editing events were similar for two different *DPH* genes (independent of the target sequence or chromosomal location), which indicates that the optimization parameters identified with this method can be generalized.

## Introduction

Gene-editing technologies, which are applicable in science as well as medicine^1^, include the use of zinc-finger nucleases (ZFNs^2–4^), transcription activator-like effector nucleases (TALENs^4–7^) and the RNA-guided CRISPR/Cas9 system^1,8–10^. The last approach is a tool that has recently emerged as the predominant choice for gene editing. CRISPR/Cas9 technology is highly specific, easy to design and generate, and well-suited for application in a variety of cell types and organisms. The target gene specificity of the nuclease Cas9 is conferred by small guide RNAs (gRNAs, usually 20mers) complementary to the sequence to be edited within the target gene. In contrast, the specificity of ZFNs and TALEN is conferred by engineered protein domains that recognize specific target sequences. Therapeutic effects can be achieved using genome editing, via the correction or inactivation of deleterious mutations, introduction of protective mutations, supplementation of transgenes and/or disruption of viral DNA^11^. The first therapeutic genome editing approach (using ZFN) addressed CCR5 in autologous CD4 T-cells of HIV patients^12,13^. The progress of therapeutic gene editing in various applications is at the preclinical stage, in addition to one phase 1 trial^11,13–17^.

Effective and robust methods for the characterization and comparison of editing technologies are essential for applications in R&D and the development of editing-based therapies. Such evaluations comprise analyses and comparisons of the efficacy as well as the specificity of target gene destruction and productive transgene integration. These aspects are particularly crucial for the safe and effective clinical translation of editing technologies^12^. Using first-generation Cas9 editing approaches, off-target modifications occur at significant rates^18–23^. Optimization of gene-editing systems is therefore desirable to reduce off-target effects while maintaining or enhancing on-target efficiency.

A prerequisite for optimizing gene editing is the reliable and robust detection and differentiation of mono- and bi-allelic gene inactivation as well as nonspecific and targeted integration events. Existing methods, such as the determination of phenotypes caused by insertions (e.g., drug resistance) or a lack of phenotypes (gene inactivation) or sequencing approaches, frequently do not differentiate mono- and bi-allelic inactivation. Moreover, existing technologies rarely address the genetic composition of individual cells and may not be based on large numbers of individual gene-edited cells to allow robust statistical analyses.

Here, we describe a simple and robust approach for characterizing gene-editing events. A combination of Diphthamide biosynthesis protein encoding gene (DPH) inactivation, diphtheria toxin (DT) treatment and puromycin (PM) selection allows the determination of gene-editing efficacy in very large numbers of individual cells. The method differentiates mono- and bi-allelic gene inactivation and indicates site-specific integration. The simplicity and robustness of the method facilitate the optimization of gene-editing procedures as well as the identification and comparison of gene-editing modulators.

## Results

### Determination of target gene inactivation and resistance cassette integration via a combination of diphtheria toxin and puromycin selection

**Figure 1:**
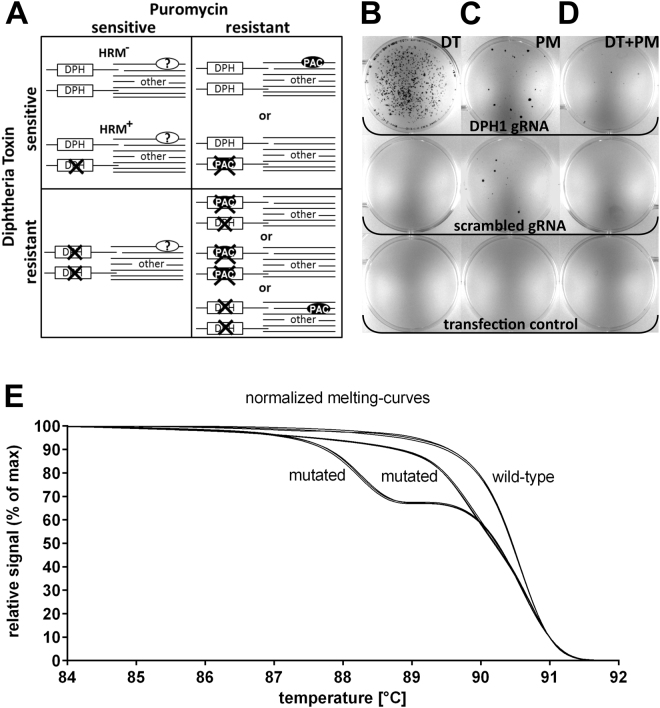
Determination of DT and/or PM resistance combined with HRM-PCR to quantify mono- vs bi-allelic gene inactivation and cassette integration events. (**A**) Overview of various repair outcomes and conferred resistance that can be distinguished by assessing resistance to DT and/or PM. Each box indicates 2 DPH1 alleles on the left and ‘other’ undefined chromosomal loci on the right. Crosses indicate gene inactivation, and HRM^+^ reflects detection of mono- or bi-allelic DPH1 sequence deviations as described in (**E**). Cassette insertion events are indicated with a solid ‘PAC-ellipse’, inserted either at DPH1 or elsewhere in transcription-enabled locations. Solid PAC-ellipses represent expressed Pac. Open ‘?-ellipses’ represent insertion events at positions that do not enable expression; these events cannot be detected by assessing PM resistance. (**B**–**D**) MCF7 cells were transfected with a CRISPR/Cas9 expression construct and a donor plasmid that integrated the pac resistance cassette in DPH1. (**B**) Cells were exposed to DT at concentrations that are lethal to cells carrying functional DPH1. In surviving colonies, all DPH1 gene copies are inactivated. Colonies that retain functional DPH1 are killed by DT. DT^r^ colonies emerge only upon treating cells with DPH1 gRNA without nonspecific background in cells exposed to control guides. (**C**) 96 hours after transfection, cells were exposed to PM at concentrations that are lethal to cells without pac. The surviving colonies carry at least one pac expression cassette and emerge in higher numbers in the presence of DPH1 gRNA compared with scrambled gRNA. The scramble guide that we applied (20mer, GCACTACCAGAGCTAACTCA) does not correspond to any specific human gene. (**D**) Simultaneous PM & DT selection reveals cells in which all DPH1 alleles are inactivated, and at least one pac cassette is integrated. (**E**) MCF7wt, MCF7wtko with one wild-type and one inactivated allele, and cells in which both alleles were inactivated were subjected to HRM-PCR spanning the target region. Cells harbouring at least one modified allele are differentiated from wt cells based on deviant melting curves. The method does not differentiate cells in which one allele is modified from cells carrying modifications on both alleles. Curve-shape analyses cannot distinguish between wt-wt and rare events potentially consisting of two identical modified alleles. However, without any exceptions, all DT-resistant cells that we analysed displayed HRM curve-shape deviations. Thus, identical modifications in both alleles (via potential dominance of particular indel types) may occur, but we did not observe any in our analyses, indicating that such events are rare under the applied methodology.

DT ADP-ribosylates diphthamide and thereby inactivates eukaryotic translation elongation factor 2 (eEF2), which irreversibly stalls protein synthesis and kills cells^24^. Diphthamide is a histidine modification placed on eEF2 via diphthamide synthesis gene-encoded enzymes, including *DPH1*. Complete bi-allelic inactivation of *DPH1* in MCF7 cells prevents the synthesis of the toxin target diphthamide, which renders cells resistant to DT^25^. Thus, inactivation of all copies of *DPH1* generates a ‘DT resistance’ (DT^r^) phenotype. *DPH1* gene inactivation as a consequence of *DPH1*–targeted gene editing can occur due to non-homologous end-joining events. In combination with a donor plasmid containing a promoter-less expression cassette encoding the enzyme puromycin N-acetyltransferase (Pac) flanked by *DPH1* homology arms, *DPH1* gene inactivation can result from the homology-directed repair of DNA double-strand breaks (and *pac* insertion). Thus, DT^r^ occurs upon inactivation of both *DPH1* alleles via either mechanism or via a combination of the two. Bi-allelic *DPH1* gene inactivation combined with homology-directed repair and *pac* expression cassette (PAC) integration into at least one allele leads to DT-PM double resistance (PM^r^ DT^r^). *Pac* insertion into one *DPH1* allele without inactivation of the other generates cells that are PM resistant but DT sensitive (PM^r^ DT^s^). The same phenotype results from cassette integration in off-target positions of the genome that enable *pac* expression (the 5′ homology arm of the *DPH1*-*pac* cassette might support transcription even though *pac* lacks its own promoter). Cells with genomic *pac* insertions at positions that do not enable expression of the cassette remain PM sensitive (PM^s^) and cannot be detected by assessing PM resistance. Figure 1A shows possible genomic events leading to the four phenotypes analysed via DT and/or PM selection: PM^s^ DT^s^; PM^r^ DT^s^; PM^s^ DT^r^; and PM^r^ DT^r^.

### Diphtheria toxin resistance assays and HRM-PCR to quantify and differentiate mono- and bi-allelic *DPH1* gene inactivation

The frequency of the DT^r^ phenotype can be detected in a robust manner by counting toxin-resistant colonies. Exposure of cells (following co-transfection with the CRISPR/Cas9/gRNA-encoding plasmid and the *pac* donor plasmid) to lethal doses (2 nM) of DT eliminates all cells that harbour at least one functional copy of the *DPH1* gene. Colonies develop only from cells in which both *DPH1* genes are inactivated (an example is shown in Figs 1B–D and S2). As the presence of one remaining functional *DPH1* allele is sufficient for toxin sensitivity, all *DPH1* alleles must be knocked out in DT^r^ cells. Cells in which only one allele is modified can be identified via high resolution melting (HRM)-PCR assays on clones derived from individual cells (Fig. 1E). This technology is based on the amplification of a genomic locus at which sequence alterations are expected, followed by recording melting curves. Modified and wild-type amplicons can be discriminated based on their melting profiles at the resolution of a single nucleotide exchange, a technology that was originally devised to diagnose single nucleotide polymorphisms or detect mutations (see Methods section for details^26,27^). Target sequence modifications consequently also alter the melting temperature of *DPH1* PCR fragments compared with that of the wild-type fragment, which generates differences in melting temperatures and, hence, bi-phasic HRM profiles. Nuclease-mediated gene inactivation events occur independently in different alleles and are therefore rarely identical in both alleles. Thus, one would expect not only ‘wild-type-mutated’ combinations but also cells with complete (bi-allelic) gene inactivation to display bi-phasic HRM profiles. In fact, all of the DT^r^ colonies that we assessed via HRM-PCR displayed deviations of the melting curve shape, which indicates that identical inactivation events in both alleles occur infrequently. Determination of the ‘toxin-resistant’ phenotype in cells subjected to HRM-PCR therefore differentiates between mono-allelic and bi-allelic (identical and non-identical) DPH1 target gene inactivation events.

### PM resistance allows detection and differentiation of specific and non-specific integration events

The *pac* integration cassette is flanked by target gene-specific homology arms (Suppl. Figure S1). Integration via homology-directed double-strand break repair results in target gene promoter-driven *pac* expression, conferring PM resistance^28^. Thus, *pac* integration is detected and quantified via PM resistance assays in a similar manner to that described for DT^r^ colonies: cells that were co-transfected with the CRISPR/gRNA-encoding plasmid and the *pac* donor plasmid were treated with lethal doses (500 ng/mL) of PM to eliminate all cells that lack *pac* expression (Fig. 1C). In contrast to DT^r^, which results only from specific and complete bi-allelic target gene inactivation, PM^r^ may occur independent of the position of integration as long as *pac* integrates into transcription-enabling loci. *Pac* expression may also occur upon integration into loci that, by themselves, are not transcriptionally active but may generate promoter activity in combination with the homology arm located upstream of *pac* (the 5′-*DPH1* arm may contain such sequences; see Suppl. Figure S1 legend for details). Non-targeted integration at positions that do not support expression will not generate PM^r^ colonies and is not detected in our assays. PM^-^ resistance assays therefore provide conservative (underestimated) estimates of non-gRNA-targeted integration events. The frequency of site-specific *versus* non-specific transcription-enabled integration is examined by comparing double-resistant DT^r^+PM^r^ colonies and PM^r^ colonies (Fig. 1D).

### Comparison of CRISPR-Cas9-mediated *DPH1* inactivation and targeted integration events

**Figure 2:**
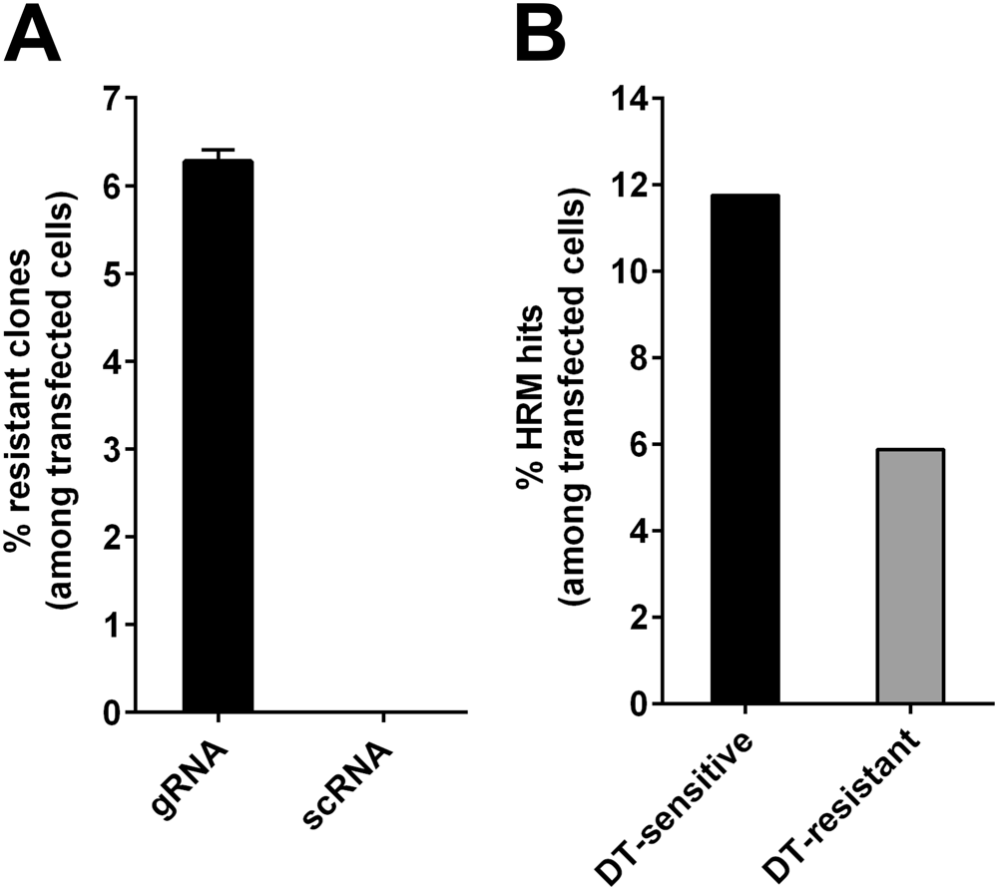
HRM-PCR and/or DT-selection of MCF7 cells transfected with the DPH1 gene-specific CRISPR/Cas9 expression construct and pac donor plasmid. Values are displayed as the % transfected cells. (**A**) DT^r^ colonies occur only when matching DPH1 gRNA is used; no colonies emerge in untreated cells or in cells that receive scRNA. Mean values +/− SEM are shown. (**B**) HRM-PCR reveals the frequency of cells that harbour DPH1 modifications on one or both alleles. Subsequent DT sensitivity assays show that mono-allelic hits (toxin sensitive & HRM positive) occur twice as frequently as inactivation of both alleles (HRM positive & toxin resistant).

To compare the frequencies of target-specific inactivation and integration and off-target integration, plasmids encoding *DPH1*-specific CRISPR/Cas9 constructs (Suppl. Figure S1) were transfected into MCF7 cells. These cells were subsequently subjected to HRM-PCR and colony count assays to measure DT and PM resistance, as described above. The results of these assays are summarized in Fig. 2, and individual datasets are available in Suppl. Table S1. Figure 2A shows that complete inactivation of the *DPH1* gene, indicating functional loss of all *DPH1* alleles, occurred at a frequency of ~6% of all transfected cells (2.5% of all cells, considering a transfection efficiency of 40%, Suppl. Table S1). *DPH1* inactivation showed absolute dependency on the matching gRNA sequence: scrambled control RNA (scRNA) did not generate any DT^r^ colonies. A comparison of the frequency of HRM hits with the occurrence of DT^r^ colonies is shown in Fig. 2B. These analyses revealed that mono-allelic gene inactivation (toxin sensitive HRM-hit) occurred twice as frequently as inactivation of both alleles (DT^r^ cells).

**Figure 3:**
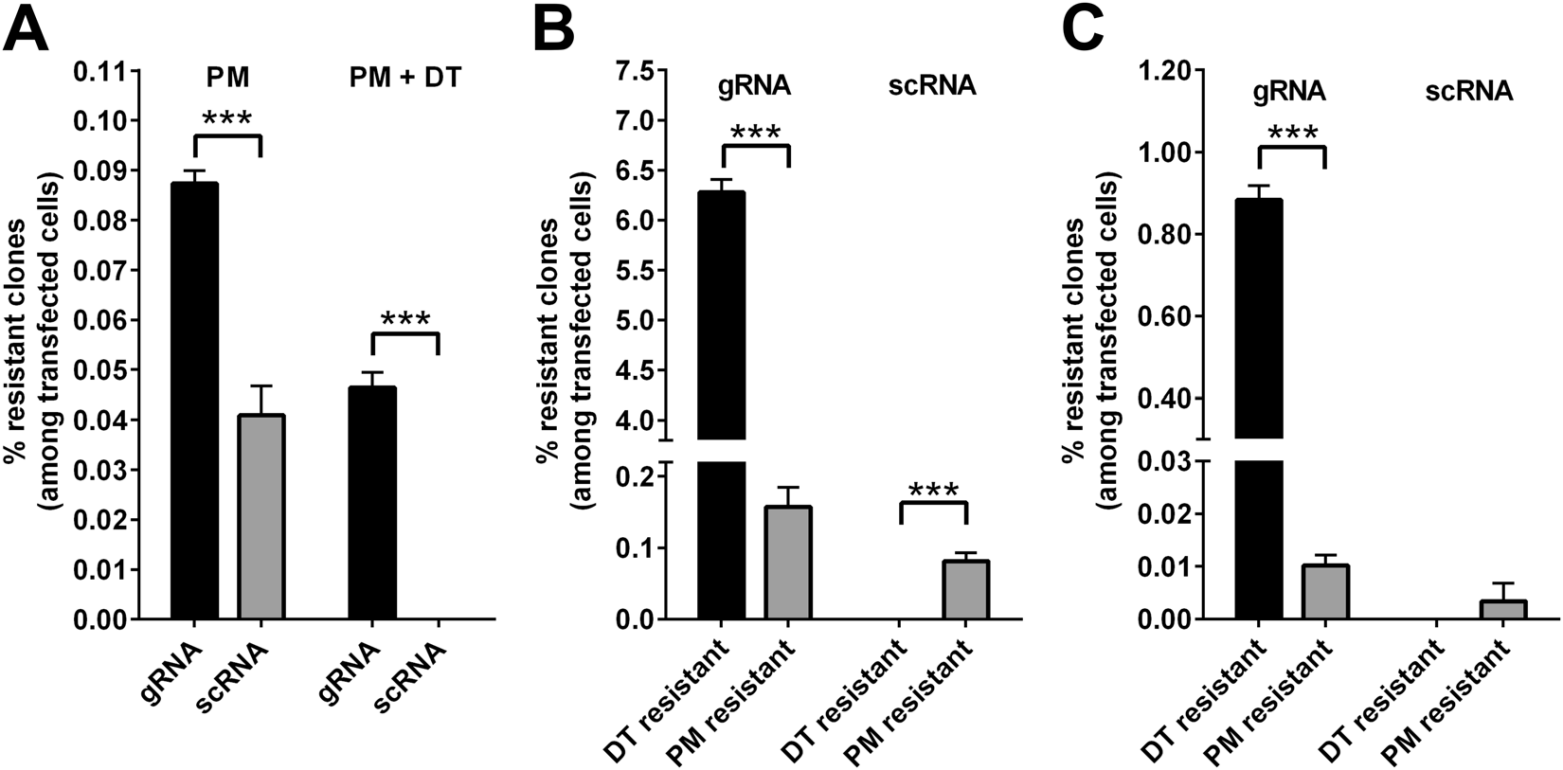
PM and/or DT selection of MCF7 cells transfected with DPH gene-specific CRISPR/Cas9 expression constructs and pac donor plasmids. Mean values + SEM are shown (n = 4, ***p < 0.001). (**A**) PM selection generates resistant colonies at a 2-fold higher frequency when DPH1 gRNA is used compared with scRNA. Combining PM selection and DT selection reveals the frequency at which the pac cassette becomes integrated in cells in which both DPH1 alleles are inactivated. DPH1 gRNA generates clones with PM-DT double resistance. scRNA generates only PM^r^ colonies and no DT^r^ colonies. (**B**) Comparison of the frequency of DT^r^ (both DPH1 genes inactivated) colonies and PM^r^ (pac integration at DPH1 or at another site) colonies. The position or zygosity of pac integration cannot be determined. (**C**) MCF7 cells transfected with DPH2-specific gRNA and donor DNA were subjected to PM and/or to DT selection. The absolute numbers of gRNA- as well as scRNA-mediated editing events are reduced for DPH2 compared with DPH1. The efficacy of targeted inactivation and integration may be due to differences in the sequence of the gRNA and homology arms and/or target gene accessibility. Reduced ‘efficacy’ of scRNA-mediated integration is a consequence of sequence features within the different homology arms of the pac cassette, as the scRNA was identical in the DPH1 and DPH2 editing experiments.

Figure 3 shows a comparison of the frequency of DT^r^ and PM^r^ colonies. Inactivation of both *DPH1* alleles (Fig. 3B) occurred with 30–50-fold higher efficacy than cassette integration events that enable *pac* expression and generate PM resistance (Fig. 3B). Compared with *DPH1*-specific gRNA, scRNA generated 2-fold fewer PM^r^ colonies under otherwise identical conditions, which reflects integration events that enable *pac* expression. Integration events in genomic regions that do not lead to *pac* expression cannot be detected by our assay. It is therefore likely that the number of random integration events is greater than the number of PM^r^ colonies. The position of *pac* integration for individual clones cannot be determined via mere determination of colony counts. Preferential gRNA-mediated integration at the gRNA-defined target gene can nevertheless be deduced by comparing the frequency of DT^r^, PM^r^, and DT^r^+PM^r^ double-resistant colonies (without the need for normalization to the transfection efficacy or scRNA controls): transfection 40,000 cells with Cas9/*DPH1*-gRNA + *pac* donor DNA results in the generation of 946 (2.4%) DT^r^ colonies and 24 (0.06%) PM^r^ colonies (Suppl. Table S1). If the two events are unrelated, the probability of observing DT^r^+PM^r^ double-resistant colonies would be 2.4% × 0.06% = 0.00144%, which translates to an expectation of ≤1 DT^r^ + PM^r^ double-resistant colony among 40,000 cells if gene inactivation and *pac* integration are unrelated events. Our observation of 12 DT^r^ + PM^r^ double-resistant colonies among 40,000 transfected cells therefore indicates a high degree of (preferential) targeted integration at the *DPH1* locus. Thus, Cas9/*DPH1*-gRNA-mediated integration preferentially occurs at the *DPH1* gene. In accordance with preferential integration in the *DPH1* gene, many of the PM^r^ colonies obtained using the *DPH1* guide were DT resistant (Fig. 3A). In contrast, none of the PM^r^ colonies obtained using scRNA were resistant to DT. Thus, Cas9-mediated gene inactivation (including that of both alleles) occurs highly specifically and with a much higher frequency than targeted *pac* integration (Fig. 3B).

### The quantification of gene editing works with another target gene, *DPH2*

Are the results obtained thus far a general feature of CRISPR/Cas9-mediated editing or specific to the *DPH1* gene? To address this question, we applied an identical approach for Cas9-induced modification of the *DPH2* gene. *DPH2* encodes a different enzyme with a different sequence on a different chromosome but is also essential for diphthamide synthesis. *DPH2* deficiency renders cells resistant to DT in the same manner as *DPH1* deficiency^25^. Thus, the assay principles developed to characterize *DPH1* modification can also be applied to analyse *DPH2* modification. The results of *DPH2* editing followed by the assessment of DT and PM resistance (with a *pac* insertion cassette that contains *DPH2* homology arms) are displayed in Fig. 3C: in line with our observations for *DPH1*, bi-allelic *DPH2* inactivation events were observed at a higher frequency than integration of the *pac* expression cassette, showing a fold change of a similar magnitude (~90-fold higher inactivation of *DPH2* than integration of the *pac* expression cassette). The absolute numbers of editing events were reduced for *DPH2* compared with *DPH1*, possibly due to the different sequence composition of the gRNA and homologous arms and/or the accessibility of the *DPH2* locus. The differences in the absolute numbers of PM-resistant colonies between *DPH1* and *DPH2* editing may also be due to potential promoter activity on the 5′ homology arm of the DPH2-*pac* cassette. The *DPH1* 5′ homology arm encompasses the immediate 5′ region of the *DPH1* gene, making it likely to contain some form of minimal promoter. Thus, insertion of the *DPH1*-*pac* cassette may lead to *pac* expression without a strict requirement for insertion behind active promoters (legend to Suppl. Figure S1B). However, the relative efficacy (compared with scRNA) was similar for *DPH2* and *DPH1*. Inactivation was strictly dependent on the presence of cognate gRNA. Cassette insertion events that enable *pac* expression occurred more frequently when *DPH2* gRNA was used than when scRNA was used (comparing the frequency of DT *vs* PM + DT resistance, see calculation above). The similarity of the *DPH1* and *DPH2* editing results indicates that the general findings obtained using this assay system will likely also apply to other genes.

### Comparison and optimization of the Cas9 gene-targeting complex: gRNA length

**Figure 4:**
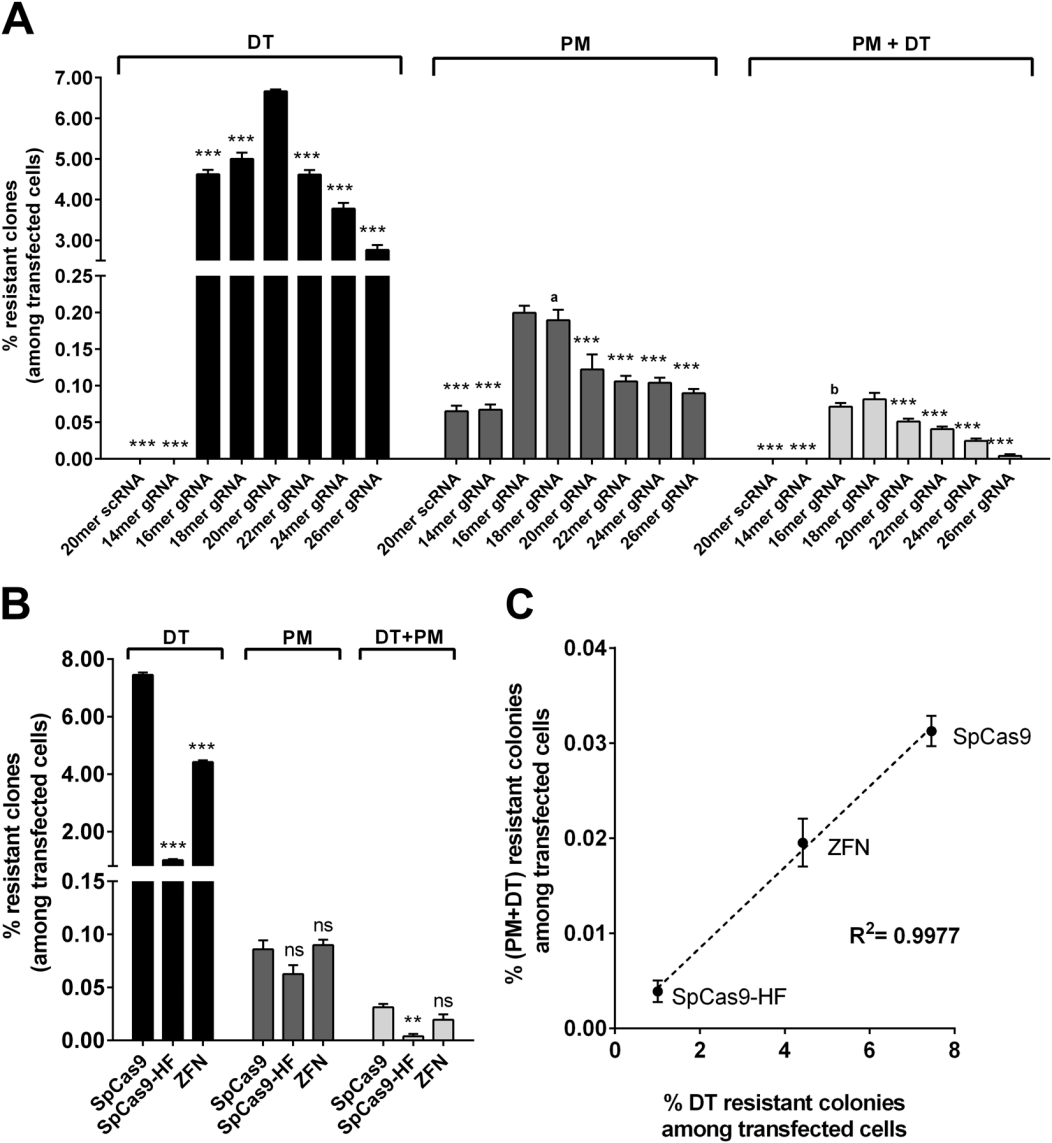
Optimization of gene editing: influence of gRNA length and editing enzymes on efficacy and specificity. (Transfection control shows neither DT^r^ nor PM^r^ colonies.) Mean values + SEM are shown (n = 4, **p < 0.01, ***p < 0.001). MCF-7 cells transfected with DPH1-specific Cas9 constructs were subjected to PM and DT selection using gRNAs of different lengths (A) or different enzymes (B&C). (**A**) gRNA length affects gene inactivation and integration frequencies. Statistical evaluation of the differences was performed by setting the gRNA with the maximum value of resistant clones for each group (i.e., DT; PM, DT+PM) as a comparator in relation to which the other gRNAs were set. These comparators were as follows: 20mer for DT; 16mer for PM, 18mer for DT+PM. a/b: no significant difference to comparator value but significant to respective 20mer gRNA value (p < 0.01) (**B**) Total number of DT^r^, PM^r^ or DT^r^ PM^r^ colonies under DPH1 editing approaches using 20mer gRNA (CRISPR/Cas9) or designed ZFN. The values are compared to the SpCas9 treatment of the respective group (DT, PM, DT+PM). (**C**) Ratio of site-specific integration events/total target gene inactivation events (DT^r^ PM^r^)/DT^r^.

Because the outcomes of the *DPH1* and *DPH2* gene-editing experiments were comparable, it can be assumed that our method identifies optimized editing parameters that can be generally applied to many other genes. Figure 4 shows how gene inactivation as well as the integration efficacy and specificity of Cas9 gRNAs of different lengths can be assessed and compared. All of the applied gRNAs targeted the same stretch of sequence within *DPH1* but varied in length from 14 to 26 bases (Fig. 4A, details of gRNAs in Suppl. Figure S1). DT^r^ colony numbers were recorded to reflect target gene-specific complete (bi-allelic) inactivation. Simultaneously, the numbers of PM^r^ and of DT^r^+PM^r^ double-resistant colonies were assessed to monitor cassette integration. As expected, gRNA length influenced the efficacy of gene inactivation, with 20mers conferring the maximal *DPH1* inactivation efficacy. Shortening the complementary stretch to 18 or 16 bases or extending it up to 26 bases retained significant specific gene inactivation functionality, albeit with a decreased efficacy compared with the 20mer. Reducing the complementary stretch within the gRNA to less than 16 bases (14mer) decreased *DPH1*-inactivating functionality to below detection levels. The integration efficacy (assessed by counting PM^r^ events) was also influenced by gRNA length. Guides smaller than 16mers (14mers) generated few PM^r^ colonies, not exceeding scrambled control background levels. Targeted integration was observed for 16mers, 18mers, 20mers, 22mers, 24mers and 26mers, with an optimum overall insertion efficacy being achieved with 16–18mers. No gain in efficacy was achieved for 22–26mer complementary stretches; in fact, stretches longer than 20mer gRNAs reduced the overall number of insertion events. The ratio between integration events (PM^r^) and inactivation events (DT^r^) can be calculated as an ‘indicator’ to identify conditions in which integration occurs with the fewest gene inactivation events. Such conditions may be favoured if one desires integration without inflicting excessive non-productive target gene damage. Low values (e.g., few PM^r^ relative to DT^r^ colonies) reflect inefficient integration in relation to simultaneously occurring inactivation events. High values (more PM^r^ and/or relatively decreased numbers of DT^r^ colonies) reflect more efficient integration. We observed the highest insertion-per-inactivation values for 16–18mers (PM/DT 16mer = 0.0431; PM/DT 18mer = 0.0379) and a significant drop for guide RNAs containing 20 complementary bases (PM/DT 20mer = 0.018) or more (p-value 18mer *vs*. 20mer = 0.0017; unpaired, two-tailed Student’s t-tests), which indicates that 20mers are quite efficient for targeted gene inactivation (in agreement with previous observations^8,29–32^). Shorter guides increase the frequency of insertion events (PM^r^ colonies) as a consequence of both targeted and nonspecific integration.

### Efficacy and specificity of different gene-editing approaches: enzymes

We compared gene inactivation and integration events and the efficacy and specificity of different variants of RNA-guided Cas9 as well as ZFN-mediated gene editing. The length and composition of gRNA were kept constant (*DPH1* 20mer), and three different editing enzymes were applied: (i) ‘SpCas9’ specifies the Cas9 nuclease from *Streptococcus pyogenes*, which can be considered the current standard application^1,33^; (ii) SpCas9-HF1 is an engineered variant of SpCas9 with reduced nonspecific DNA binding and off-target activity and, hence, a proposed higher fidelity and specificity^19^; and (iii) a ZFN-editing entity that recognizes target sequences via designed zinc finger-mediated protein-nucleic acid interactions^34,35^.

In the same manner as for gRNA analyses, DT^r^ colonies were recorded to reflect targeted gene inactivation, and PM^r^ colonies were recorded to monitor cassette integration (Fig. 4B, Suppl. Table S3). In comparisons of the overall efficacy of gene inactivation and cassette integration, the highest values for both parameters were observed using CRISPR/SpCas9. CRISPR/SpCas9-HF diminished targeted gene inactivation events to less than 20% of the number of DT^r^ colonies compared with CRISPR/SpCas9. The frequencies of PM^r^ (integration) and DT-PM double-resistant colonies (integration with targeted gene inactivation) were also reduced. Application of ZFN reduced the number of DT^r^ colonies under otherwise identical conditions to less than 60% of the events observed using CRISPR/SpCas9. The efficacy of ZFN-targeted inactivation was therefore ~2-fold reduced compared with SpCas9 and ~2–3 fold better than that of the engineered SpCas9-HF1. The frequency of PM^r^ colonies did not significantly differ between CRISPR/SpCas9 and ZFN. Double-resistant colonies (cassette integration with simultaneous gene inactivation) were somewhat (30%) reduced using ZFN compared with CRISPR/SpCas9. Calculation of the ratio of DT^r^ (target gene inactivation) to DT+PM double-resistant (targeted integration) colonies takes overall efficacy out of the equation, indicating that CRISPR/SpCas9, CRISPR/Cas9-HF, and ZFN generated the same level (~4 × 10^−3^) of targeted integration events per bi-allelic gene inactivation event (Fig. 4C).

### Influence of DNA repair modulators on gene-editing efficacy and specificity

**Figure 5:**
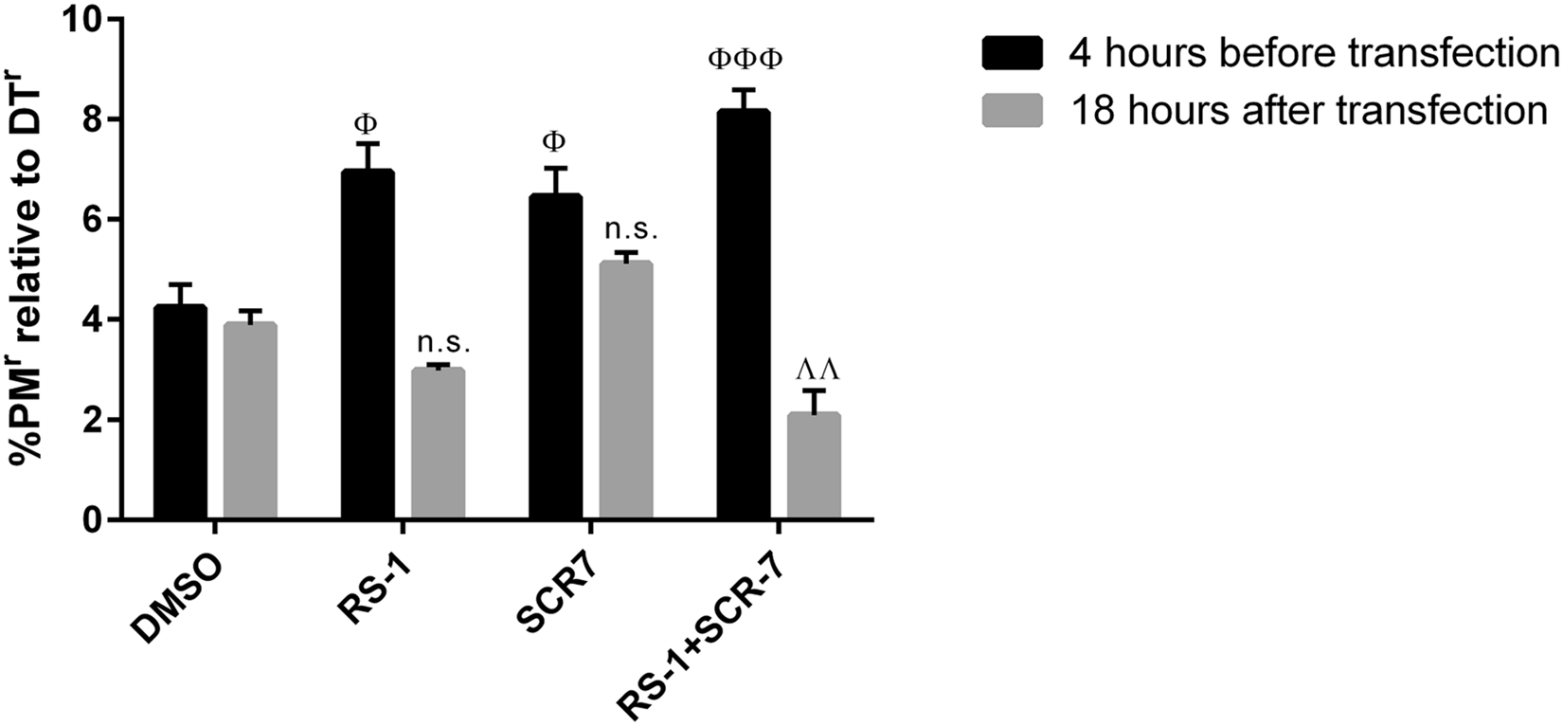
Influence of DNA repair-modulating agents on gene editing. MCF-7 cells were transfected with plasmids encoding 20mer gRNA, SpCas9 and pac as described previously. The solvent control (DMSO), HDR-modulating agent RS-1 (8 µM) and NHEJ-modulating SCR7 pyrazine (1 µM) were added either 4 hrs before or 18 hrs after transfection. DT or PM selection was initiated 72 hrs after transfection. The percentage of PM^r^ colonies (integration) relative to DT^r^ colonies (cleavage) is shown. The values are compared to the DMSO control the respective addition time-point. Mean values + SEM are shown (n = 4, ^Φ^p < 0.05, ^ΛΛ^p < 0.01, ^ΦΦΦ^p < 0.001).

Colony assays for quantifying DT^r^ and PM^r^ cells following DPH gene editing can also be used to address the influence of compounds that modulate DNA repair. Activators of homology-directed repair (HDR) and inhibitors of non-homologous end joining (NHEJ) modulate gene-editing events and increase integration efficacy^36,37^. To demonstrate the suitability of our technology for determining the effect of DNA repair modulators on the efficacy and specificity of editing, CRISPR/SpCas9/*DPH1*gRNA (20mer) editing and *pac* integration assays were combined with such compounds, and the influence was quantified. The DNA ligase IV inhibitor SCR7 pyrazine was applied either 4 hrs before transfection (‘early addition’) or 18 hrs after transfection (‘late addition’) of the gene-editing constructs, and exposure was continued until 96 hrs after transfection. We used the HDR-active pyrazine derivate of SCR7 in our experiments (see Methods section). Similarly, the RAD51 modulator RS-1 (RAD51-stimulatory compound 1) was added to stimulate HDR. Both compounds were applied at doses that had no effect on the growth or viability of MCF7 cells (see Methods section): 1 µM for SCR7 pyrazine, 8 µM for RS-1, and 1 µM + 8 µM for SCR7 pyrazine + RS1. Compared with the DMSO-treated control, the addition of RS-1 increased the number of PM^r^ colonies ~2-fold (Suppl. Table S4). To quantify the effect on the overall integration efficacy, the percentage of PM^r^ colonies (gene integration) relative to DT^r^ colonies (gene inactivation) was calculated (Fig. 5). The addition of RS-1 at an early time point led to a significantly higher integration efficacy; however, it did not affect the integration efficacy upon late addition (18 hrs after initiation of editing). Thus, choosing the appropriate (early) time point for RS1-mediated HDR stimulation is important for the enhancement of productive editing, confirming HDR to be a driver of targeted cassette integration. To a similar degree, early application of SCR7 pyrazine significantly increased the relative number of integrations (Fig. 5 and Suppl. Table S4), which confirms previous observations of enhanced productive gene editing upon SCR7 pyrazine administration^37^. When both compounds were used, the ratio of PM^r^ relative to DT^r^ was 8.1%, compared with 6.5% (only SCR7 pyrazine) or 6.9% (only RS-1). However, these differences/increases were not significant (p = 0.39 *vs* RS-1 alone), which is in line with previous observations^38,39^.

## Discussion

**Figure 6:**
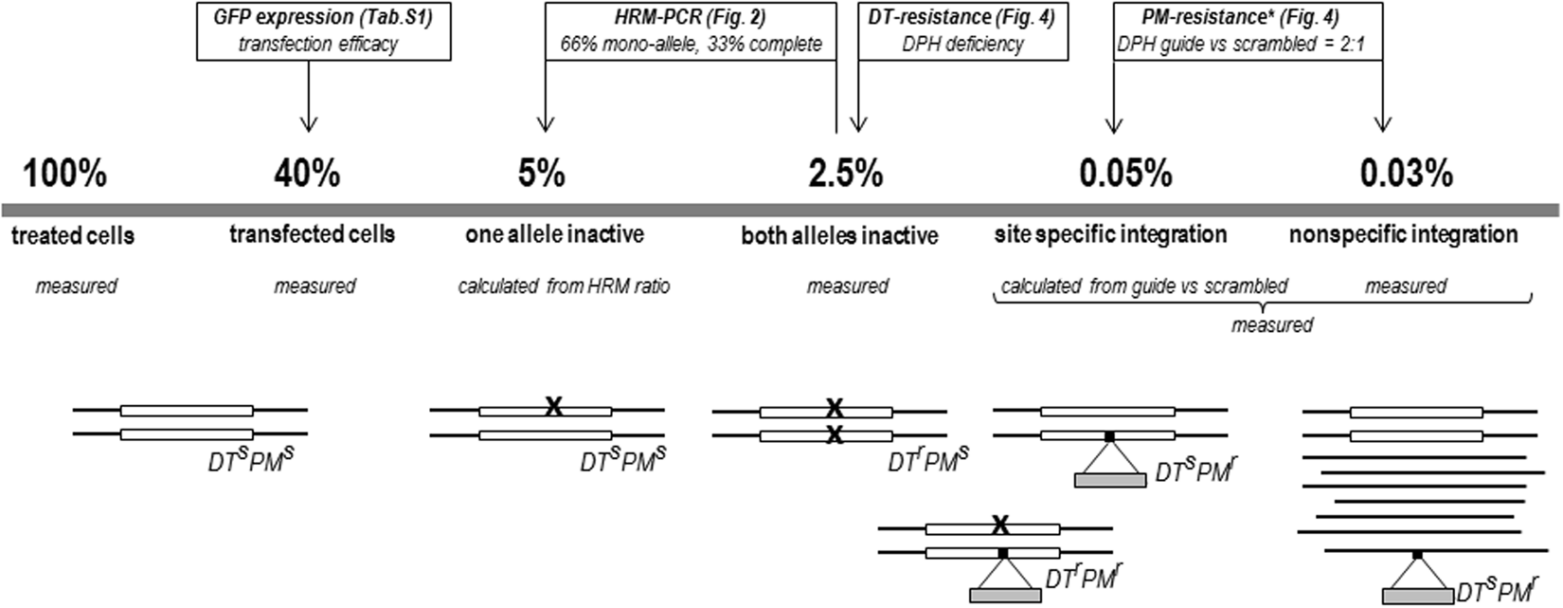
Frequency of CRISPR/Cas9-mediated gene-editing events. The average event frequencies obtained via determination of the numbers of PM^r^, DT^r^ and double-resistant cells upon CRISPR/Cas9 editing of DPH1 with 20mer gRNA are shown. DT-sensitive mono-allelic DPH1-edited cells are quantified based on HRM-PCR results indicating a 2:1 ratio of mono- vs bi-allelic inactivation events. Site-specific integration can result in DT^s^ PM^r^ colonies (integration at DPH1 with the 2^nd^ allele unaltered) as well as double-resistant DT^r^ PM^r^ colonies (integration and bi-allelic DPH1 inactivation). *PM^r^ colonies occurring following scRNA editing may be due to homology arm-mediated integration at the target gene (pac cassette contains homology arms) or to integration at transcription-enabling non-target sites. As integration events that do not enable transcription are not detected, the overall nonspecific integration frequency, including non-expression-enabling events, is expected to be higher than indicated.

Genome editing has emerged as a technology of utmost importance for scientific and potential therapeutic applications. Its entire potential is, however, still limited by efficacy and specificity issues of the currently applied editing approaches. The presented method enables simple and robust quantification and comparison of the efficacy and specificity of gene inactivation and donor cassette insertion events. The core principle of this method consists of inactivation of the endogenous diploid *DPH1* or *DPH2* genes, which results (provided it occurs on both alleles) in absolute resistance to DT. The additional insertion of the *pac* gene allows the determination of both targeted and non-targeted integration via the respective selection methods. Due to the simplicity and robustness of these readouts (colony counts), the method allows exact determination of mono- and bi-allelic target gene inactivation and nonspecific *versus* targeted integration events based on large numbers of individual cells (shown in Fig. 6). Furthermore (and in contrast to many existing tools^33,40–42^), mono- and bi-allelic target gene inactivation and integration events can be differentiated. Thus, simple colony counts reflect the efficacy of and ratios between productive (integration) and destructive gene editing (inactivation without integration). The results obtained by applying this method may be of particular importance in the development and optimization of gene-editing approaches, such as methods for the generation of genetically defined cell lines or organisms, and potentially also for therapeutic gene editing.

*Evidence that the method delivers ‘generalizable’ results* was obtained by comparing editing events (colony frequency) involving two different *DPH* genes. *DPH1* and *DPH2* encode different enzymes, both of which are independently essential for diphthamide synthesis. The results revealed comparable efficacies, specificities and destruction/integration ratios for the two genes, which indicates that the dependencies and parameters obtained via this method are transferrable to optimization of the editing of other genes. As a proof of concept and benchmark validation of our method, we addressed and confirmed the influence of several previously analysed parameters on gene editing, as listed below.

*The length of gRNA for CRISPR/Cas9-mediated editing* influences the efficacy of nonproductive gene inactivation as well as productive targeted integration^43–45^. In line with previous analyses^30^, our assays unambiguously demonstrate that ‘standard’ 20 mer gRNAs are effective for Cas9-mediated gene targeting, generating the highest overall gene inactivation frequency. The simplicity of our assay enables the simultaneous assessment of gRNAs of diverse lengths, revealing threshold sizes below or above which efficacy becomes compromised. One interesting observation within this context was that the best ratios between productive and destructive editing events were observed using 16–18 mer guides. Thus, 20 mers may be the preferred choice for efficient gene inactivation, while 16–18 mers are preferred if one desires integration without excessive destructive editing. Fu *et al*.^33^, tested <20 mer gRNAs in gene inactivation experiments and observed an efficacy comparable to 20 mers, with simultaneously reduced off-target effects. Their analyses were based on mono-allelic GFP gene inactivation. As their method involved only one target gene per cell, it could not address or differentiate between mono- and bi-allelic inactivation events in diploid cells and could not compare insertion events. Our approach (based on large numbers of cells and inactivation of normal chromosome-encoded human genes) demonstrated that 20 mers are more efficient mediators of gene inactivation than shorter guides. Shorter guides increase the frequency of insertion events (PM-resistant colonies) as a consequence of either targeted or nonspecific integration.

*The choice of gene-editing enzymes* *,* such as CRISPR/Cas9/gRNA or protein (e.g., ZFN)-based recognition systems and derivatives, is another factor that influences editing efficacy and possibly specificity. Our method is not restricted to the standard CRISPR/Cas9 system and can be also applied to monitor gene-editing efficacy for other gRNA-targeted Cas9 derivatives or protein-targeted approaches, such as those based on ZFN^34,35,46–50^. In the comparison of ZFN, CRISPR/Cas9 and HF-Cas9 editing, we observed the highest overall efficacy of gene inactivation and cassette integration for the ‘original’ CRISPR/SpCas9 system. Compared with this system, reduced efficacy was observed for both the ZFN and high-fidelity HF-Cas9 variant systems. In agreement with previous observations^19^, HF-Cas9 dramatically reduced scRNA-mediated (hence, most likely non-specific) integration events to below-detection limits.

*The specificity of gene editing* was assessed by comparing the frequency of colonies emerging under DT selection (bi-allelic target gene inactivation), PM selection (cassette integration) and DT+PM double selection (inactivation and integration). Target gene inactivation via CRISPR/spCas9 or HF-Cas9 occurs with ‘absolute’ dependence on gRNA specificity, *i.e*., only when applying cognate gRNAs without any scRNA background. In contrast, scRNA background was observed (as expected) when assessing PM^r^ colonies. Our colony count assays are not suited to assessing the position of *pac* integration for individual clones, which would require sequencing, involving either many cells in a population (without differentiating alleles of individual clones) or defined clones (defined allele compositions of a limited number of events). Our approach deduces the probability of targeted integration events according to comparison of the frequency of DT^r^, PM^r^, and DT^r^+PM^r^ double-resistant colonies, based on large numbers of individual colonies. This approach requires neither normalization of transfection efficacy nor scRNA controls, as all data stem from a single editing experiment assessing DT^r^, PM^r^ and DT^r^+PM^r^ double-resistant colonies. DT^r^ and PM^r^ colony numbers reflect the individual frequency (e.g., in % of transfected cells) of gene inactivation or integration, and the frequency of DT^r^+PM^r^ double-resistant colonies indicates whether (and to what degree) the two events are individual events or are ‘linked’. The ‘extremes’ of these calculations (frequency of DT^r^+PM^r^) = (frequency of DT^r^) × (frequency of PM^r^) would correspond to *pac* insertion occurring nonspecifically without gRNA involvement or all PM^r^ colonies are also being DT^r^ (frequency of DT^r^+PM^r^) = (frequency of PM^r^). In the latter case, all *pac* insertions would occur at the target gene (as the coincidence of double target gene inactivation with non-targeted insertion elsewhere is negligibly low). The degree of independence or linkage of DT^r^ and PM^r^ colonies can therefore be regarded as a measure of specificity when comparing different editing approaches or editing modulators.

*Compounds that modulate recombination* have recently been used to increase the efficacy of productive (integration) editing. Examples of such compounds include the ligase IV inhibitor SCR7 pyrazine (see Methods section for details of the compounds) for modulation of non-homologous end-joining (NHEJ) and the homology-directed repair (HDR) stimulator RS-1^36,37^. The suitability of our method for determining the effect of NHEJ- and HDR-modulating agents on gene editing allows us to compare it to available screening approaches described in the literature. The application of our method to editing in combination with these compounds confirmed all previous observations of SCR7 pyrazine- and RS-1-mediated increases in efficacy^37^. Pinder *et al*. invented a FACS-based assay that exploits the site-specific integration of a fluorescent protein. This approach detects integration within single cells, yet without addressing zygosity or quantifying off-target integration^38^. In contrast to their approach, our readout is based on the phenotype resulting from endogenous gene modification and allows the quantification of NHEJ repair as well as site-specific repair and HDR (via double selection, and the probability of co-event comparison, see above). Furthermore, our ‘colony count assays’ recapitulate the animal-based results of Song *et al*.^36^, demonstrating HR/NHEJ ratios (gene inactivation-to-integration) of below 10% as well as RS-1-mediated enhancement of HR and integration. It must be noted that in contrast to other assessment technologies^36,38^, our method permits the assessment of modulators in a simple ‘downstream-assay free’ cell culture setting and could serve as a screening or pre-selection technology before initiating *in vivo* studies. Cell-based colony count approaches are high-throughput compatible, and death *vs* survival readouts are very robust. Thus, the method can (in addition to the examples above) be used to measure and quantify editing events in the context of various additional parameters, which may include the assessment and further characterization of modulating compounds and/or the definition of active components of compounds whose activities are under discussion (e.g., SCR7 *vs* SCR7-pyrazine as a DNA ligase I/III and/or IV inhibitor,^51^). It also enables the screening of potential additional editing enhancer candidates, collections or libraries (including recombination and repair modulators), identification of the most effective mode of delivery for editing entities (mRNA, protein or DNA) as well as the composition of the donor cassette (length of insert and homology arms) for targeted insertions.

## Methods

### Cultivation of MCF7 cells and transfection of plasmids encoding gene-editing entities

MCF7 cells^52^ were originally obtained from the ATCC (Manassas, VA, USA) and maintained in RPMI 1640 medium supplemented with 10% FCS, 2 mM L-glutamine and penicillin/streptomycin at 37 °C and 85% humidity. Within a set of experiments, we used one batch of cells to ensure that the comparisons and conclusions that we made were not affected by variance in the speed of colony formation. Between the experimental sets, we thawed new cell batches to ensure that the cells did not develop genomic alterations over time. For the transfection of plasmids harbouring gene-editing constructs, 3,000,000 cells were seeded in a 10 cm-diameter culture dish and cultivated at 37 °C in a humidified 5% CO_2_ atmosphere. At 24 h after seeding, the cells were transfected with 20 µg of total DNA using jetPEI (Polyplus) according to the manufacturer’s protocol, except that an N/P ratio of 6:1 was employed. Transfection efficiency was determined 24 h thereafter via flow cytometry (FACSCalibur, BD Biosciences) of cells that were transfected with an eGFP expression plasmid^53^. Plasmids encoding CRISPR/Cas9 editing entities targeting *DPH1* (gRNA target: CAGGGCGGCCGAGACGGCCC derived from RefSeq: NM_001383) and *DPH2* (gRNA target: TCGTACACTCCGTCCAGGTC derived from RefSeq: NM_001039589, NM_001384), as well as scrambled control RNA (scRNA: GCACTACCAGAGCTAACTCA) were obtained from Origene (*DPH1*# KN221955; *DPH2*# KN201382). This system comprises one plasmid expressing gRNA under the control of a U6 promoter, Cas9 nuclease under the control of a CMV promoter, and a donor plasmid with a promoter-less *pac* expression cassette flanked by homologous arms to the target gene (*DPH1* or *DPH2*, see Suppl. Figure S1 for details). Additional *DPH1* gRNAs of different sizes (Origene) included the 14mer GGCCGAGACGGCCC; 16mer GCGGCCGAGACGGCCC; 18mer GGGCGGCCGAGACGGCCC, 22mer AGCAGGGCGGCCGAGACGGCCC; 24mer GGAGCAGGGCGGCCGAGACGGCCC and 26mer GCGGAGCAGGGCGGCCGAGACGGCCC (Suppl. Figure S1).

### Quantification of CRISPR/Cas9-mediated bi-allelic *DPH1* and *DPH2* gene inactivation

MCF7 cells in which all chromosomal copies of *DPH1* or *DPH2* are inactivated are DT resistant^25^. Thus, the occurrence and frequency of toxin-resistant cells/colonies upon gene inactivation provide a measure of the efficacy of inactivation of all gene copies. MCF7 cells were transfected as described above using (i) a GFP expression plasmid, as a transfection control; (ii) the CRISPR/Cas9 *DPH1* or *DPH2* knock-out/integration system; and (iii) knock-out/integration entities containing scRNA, as a control. After determination of the transfection efficiency, 10,000–40,000 cells were seeded in 6-well plates. RPMI medium was exchanged with RPMI medium containing DT (2 nM) 3 days after cell seeding. The medium was exchanged every 2–3 days until dead cells became detached. Between day 12 and day 14 after the initiation of toxin exposure, cells were washed 3 times with PBS and stained with ice-cold methylene blue (0.2% in 50% EtOH), followed by gentle washing under running water. Stained and fixed colonies were recorded via microscopy counting on 5 × 5 mm grid foil for orientation. The complete raw data (*i.e*., colony numbers from individual experiments) are provided in the supplementary information (Table S1).

### Detection of CRISPR/Cas9-mediated mono-allelic DPH gene inactivation

Cells in which only one *DPH1* or *DPH2* allele is modified are DT sensitive. To identify and quantify such events, high-resolution melting (HRM) PCR was applied in a similar manner as previously described^25^: 24 h after transfection, single cells were deposited in 96-well plates through FACS (FACSAria^TM^, BD Biosciences) and grown to confluency. The cells were washed with PBS and lysed by the addition of 40 µL of cell lysis buffer (Roche) per well. After 15 mins of incubation at RT on a plate shaker (Titramax 1000, Heidolph) at 750 rpm, the cell lysate was diluted 1:5 with PCR-grade H_2_O. Then, 5 µL of the cell lysate was mixed with HRM master mix (Roche) and primers spanning the gRNA target sequence. PCR and HRM were performed on the LC480 II platform (Roche) according the manufacturer’s protocol. Clones with edited target genes were identified based on melting curve deviations compared with MCF7-wt cells. Cells displaying biphasic melting curves may still possess one wt allele, or both alleles may be inactivated. Because nuclease-mediated gene inactivations are independent events in different alleles, they are rarely identical in both alleles (in our hands, all DT^r^ colonies displayed bi-melting curve-shape deviations. Differentiation between wt and two identical modified alleles by HRM is in principle also possible because the melting temperatures of wt and mutated alleles differ if only one base is changed (the principle of HRM-mediated SNP-diagnostics (ref.^26^ and^27^)). We nevertheless suggest ‘abnormal curve shape’ as a readout because this readout is simple and robust, is not influenced by potential DNA, salt or buffer content variations in cell extracts and, hence, does not require highly standardized procedures for extract preparation. Clones displaying melting curve deviations were expanded without DT or PM selection and subjected to viability analyses to discriminate between toxin-sensitive mono-allelic and resistant bi-allelic knockout cells. These assays were performed in 96-well plates containing 10,000 cells at 37 °C in humidified 5% CO_2_. At 24 hr after seeding, the cells were exposed to toxin for 72 h. Metabolic activity was assessed via the CellTiter Glo® Luminescent Viability Assay (Promega).

### Identification and quantification of CRISPR/Cas9-induced transgene integration

In addition to the Cas9 nuclease and gRNA or scRNA, the applied CRISPR/Cas9 knock-out/integration system also contained a *pac* expression cassette without a promoter to avoid transient expression flanked by homologous arms for HDR (donor DNA). Thus, detection of the integration of recombinant sequences into the genome was performed via determining the PM sensitivity of cells. The frequency of both events (gene inactivation and integration) was detected through the application of DT and PM. MCF7 cells were transfected and treated as described for the identification and quantification of gene inactivation, applying PM (500 ng/µL) or a combination of PM (500 ng/µL) and DT (2 nM). Complete data (*i.e*., colony numbers from individual experiments) are provided in the supplementary information (Table S1).

### Identification and quantification of ZFN-mediated *DPH1* gene editing

MCF7 cells in which all chromosomal copies of *DPH1* are inactivated are DT resistant^25^. Thus, the occurrence and frequency of DT^r^ colonies following ZFN-mediated gene inactivation and/or cassette integration provides a measure of the efficacy of inactivation of all gene copies. The ZFN recognition sequence (CAGGTGATGGCGGCGCTGG*TCGTA*TCCGGGGCAGCGGAGCAG, *cleavage site*) is derived from NM_001383.3 (*DPH1*-wt) and was obtained from Sigma. A *pac* integration cassette for this position was obtained from Origene. MCF7 was transfected as described above using (i) a GFP expression plasmid, (ii) the plasmid encoding *DPH1*-targeting ZFN and (iii) the *DPH1*-targeting *pac* integration cassette. After determination of the transfection efficiency, the cells were seeded in 6-well plates. For quantification of bi-allelic knock-out events, (DT^r^) 20,000 cells were seeded; 40,000 cells were seeded for the quantification of integration events (PM^r^) or double resistance. RPMI medium was exchanged with RPMI containing DT, PM or both 3 days after seeding. The medium was changed every 2–3 days. Between day 12 and day 14 after the initiation of toxin exposure, cells were washed 3 times with PBS and stained with ice-cold methylene blue (0.2% in 50% EtOH), followed by gentle washing under running water and microscopic determination of colony numbers using 5 mm grid foil.

### Quantification of the effects of HDR and NHEJ modulators on CRISPR/Cas9-mediated editing

RAD51-stimulatory compound 1 (RS-1) was applied to modulate homology-directed repair (HDR) during gene editing^36^. RS-1 (Sigma, R9782) was dissolved in DMSO to generate a stock solution of 10 mg/mL, which was diluted in RPMI medium just before application to cells. Viability (Promega CTG) assays identified a final concentration of 8 µM RS-1 as a dose that does not inflict growth-inhibitory or toxic effects on MCF7 cells (viability: 1 µM, 100%; 3.7 µM, 100%; 11 µM, 97%; 33 µM, 61%). The DNA ligase IV inhibitor SCR7 pyrazine was applied to modulate non-homologous end joining (NHEJ) during gene editing^37^. SCR7 pyrazine (Sigma, SML1546) was dissolved in DMSO to generate a stock solution of 10 mg/mL, which was diluted in RPMI medium just before application to cells. Viability (Promega CTG) assays identified a final concentration of 1 µM as a dose that does not inflict growth-inhibitory or toxic effects on MCF7 cells (viability: 0.37 µM, 100%; 1.1 µM, 100%; 3.3 µM, 97%; 10 µM, 88%). SCR7 pyrazine (1 µM final conc.), RS-1 (8 µM final conc.) or SCR7 pyrazine + RS-1 (1 µM + 8 µM final conc.) was added to MCF7 cells 4 hrs before transfection of the gene-editing constructs in the ‘early exposure’ setting. For ‘late exposure’, SCR7 pyrazine (8 µM final conc.) or RS-1 (1 µM final conc.) was added to MCF7 cells 18 hrs after transfection. In both settings, the cells were exposed to the modulators until 96 hr after transfection, i.e., ‘early exposure’ consisted of treatment for a total of 100 hrs and ‘late exposure’ for a total of 78 hrs. The system for determining the effects of DNA repair modulators consisted of MCF7 cells transfected with the CRISPR/SpCas9 constructs including *DPH1* 20mer gRNA and then subjected to DT and PM selection, as described above. The frequencies of DT^r^, PM^r^, and double-resistant colonies were recorded to reflect gene inactivation and cassette integration events.

### Statistics

Unpaired, two-tailed Student’s t-tests were performed for single comparisons between two treatments. Multiple comparisons were statistically analysed via a one-way ANOVA, followed by Tukey’s honestly different significance (HDS) post hoc test. A significant difference was defined by a p-value of <0.05. The level of significance determined using Student’s t-test or Tukey’s HDS test is indicated in graphs by one, two or three symbols (*, Λ or Φ) corresponding to p < 0.05, p < 0.01 and p < 0.001, respectively.

## Electronic supplementary material

Supplementary Information
