# Supplementary material for: Disruption of diphthamide synthesis genes and resulting toxin resistance as a robust technology for quantifying and optimizing CRISPR/Cas9-mediated gene editing
Source: Sci Rep. 2017 Nov 13;7:15480. doi: 10.1038/s41598-017-15206-x (PMC5684134; doi:10.1038/s41598-017-15206-x)
Supplement: Supplementary file 1 — Supplementary Information [file 41598_2017_15206_MOESM1_ESM.pdf]

Supplementary Information

Disruption of diphthamide synthesis genes and resulting diphtheria toxin resistance as a robust technology for quantifying and optimizing CRISPR/Cas9-mediated gene editing

Tobias Killian[1], Steffen Dickopf[1], Alexander K Haas[1], Claudia Kirstenpfad[1], Klaus Mayer[1] & Ulrich Brinkmann[1]*

[1] Roche Pharma Research and Early Development (pRED), Therapeutic Modalities - Large Molecule Research

Roche Innovation Center Munich, Nonnenwald 2, D-82372 Penzberg, Germany

* corresponding author: ulrich.brinkmann@roche.com

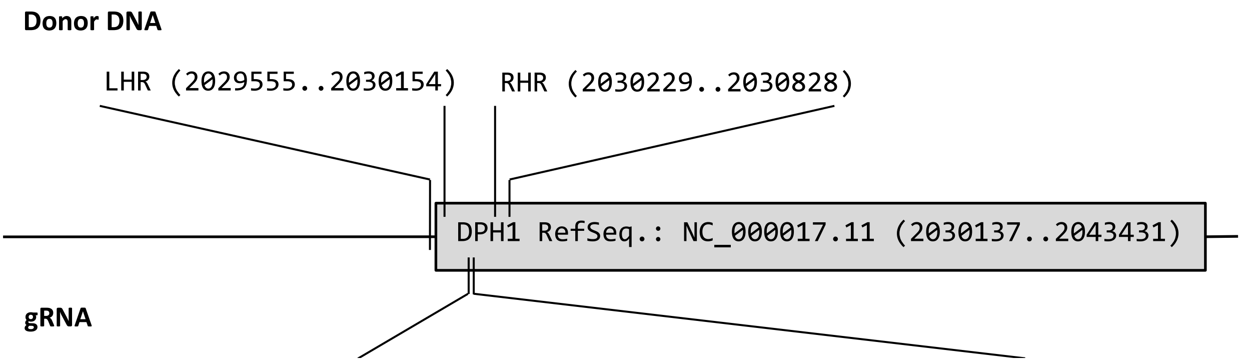


```
26mer    GCGGAGCAGGGCGGCCGAGACGGCCC (2030197..2030222)
24mer      GGAGCAGGGCGGCCGAGACGGCCC (2030199..2030222)
22mer        AGCAGGGCGGCCGAGACGGCCC (2030201..2030222)
20mer          CAGGGCGGCCGAGACGGCCC (2030203..2030222)
18mer            GGGCGGCCGAGACGGCCC (2030205..2030222)
16mer              GCGGCCGAGACGGCCC (2030207..2030222)
14mer                GGCCGAGACGGCCC (2030209..2030222)
```

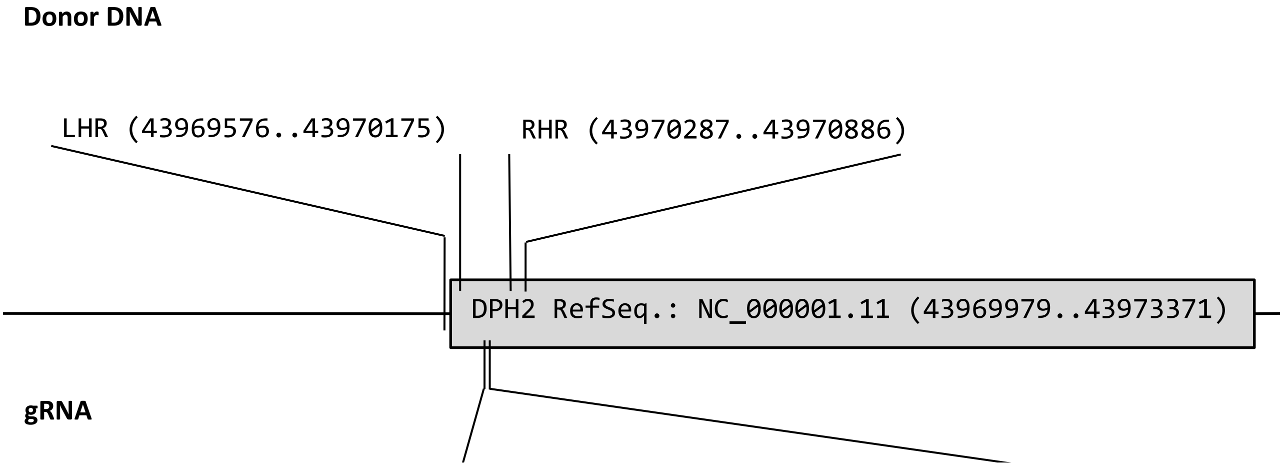


```
20mer    TCGTACACTCCGTCCAGGTC (43970276..43970257)
```

Figure S1: (A) Composition of gRNAs: *Sequence, size and exact genomic loci of DPH1 and DPH2 targeting gRNAs according to assembly GRCh38.p7 (GCF_000001405.33) for both genes; exact chromosomal position is indicated in brackets; LHR: left homologous region and RHR: right homologous region of the integration cassette on the donor plasmid. Corresponding mRNA sequences are RefSeq: NM_001383 (DPH1) and RefSeq: NM_001039589, NM_001384 (DPH2). Scrambled scRNA applied as control for DPH1 and DPH2 editing was the 20mer GCACTACCAGAGCTAACTCA which does not address any specific human gene.*

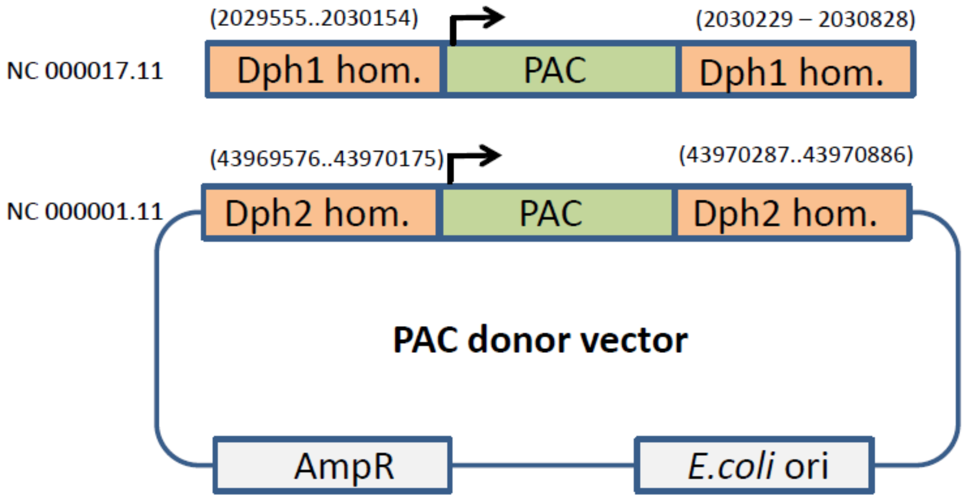

Figure S1 (B) Composition of plasmids for PAC insertion

The integration cassette encoding pac without its own promoter is flanked by sequences homologous to DPH1 or DPH2 (numbering according to assembly GRCh38.p7 (GCF_000001405.33). Pac expression occurs upon homology-directed insertion into DPH1 or DPH2 loci, respectively. Pac expression may also result from insertion into loci different from DPH1 or DPH2 that enable transcription (in combination with DPH1 or DPH2 flanking regions). It has also been brought to our attention by the reviewers that the DPH1 5'-homology arm encompasses the immediate 5' region of the DPH1 gene, making it likely to contain some form of minimal promoter. Thus, insertion of the DPH1-pac cassette may lead to expression without strict requirement of insertion behind active promoters.

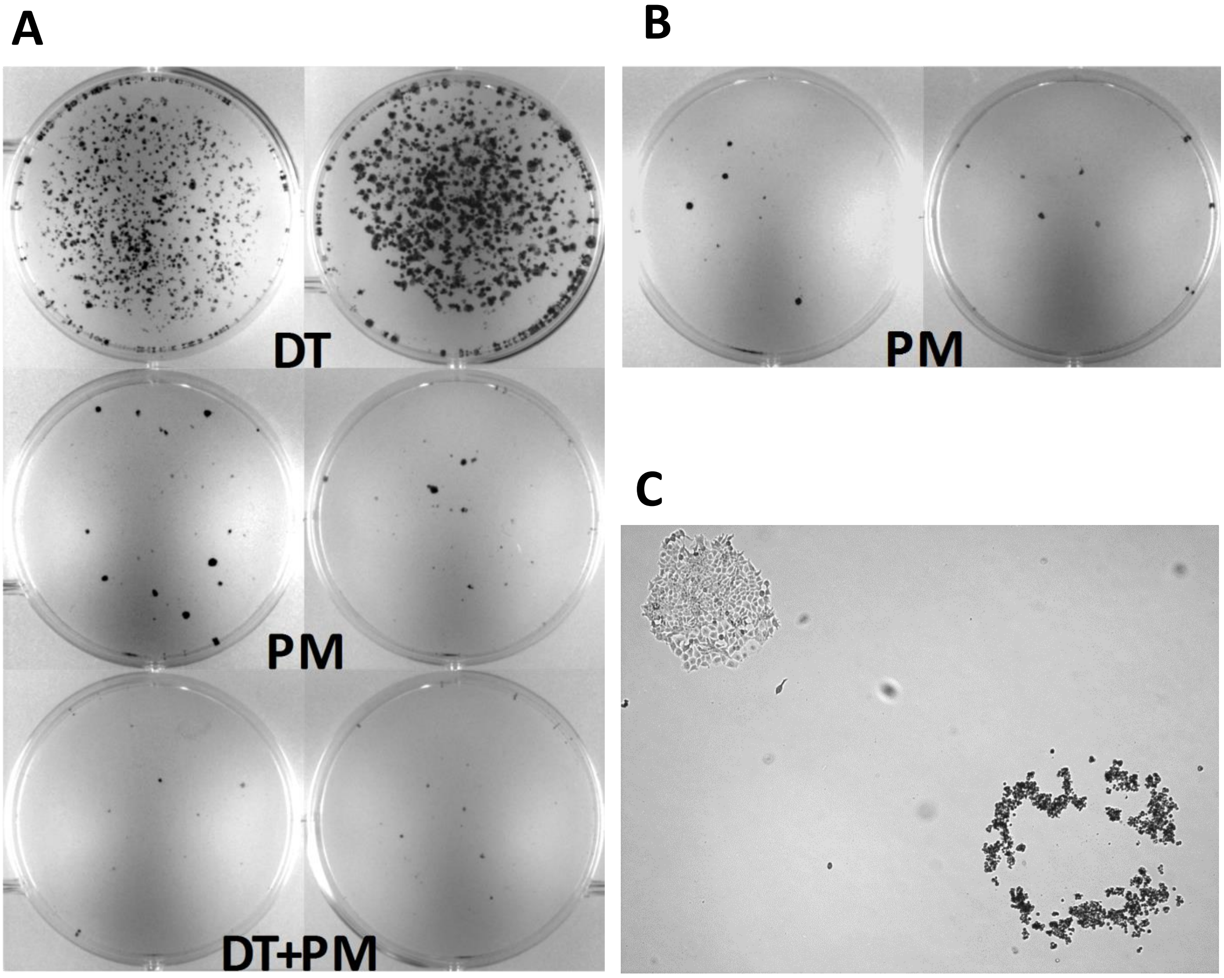

Figure S2: Images of colony quantification experiments

(A) MCF7 cells were transfected with plasmids encoding CRISPR/Cas9 constructs targeting and integrating a pac expression cassette in DPH1. 48 hours after transfection, cells were exposed to DT, PM or DT+PM respectively. (B) MCF7 cells were transfected with a plasmid encoding CRISPR/Cas9 with a scrambled gRNA and the plasmid with the pac expression cassette for integration. 48 hours after transfection, cells were exposed DT, PM or DT + PM respectively. Resistant colonies were only observed after PM selection. (C) Image of DT resistant (left) and DT sensitive (right) colony.

Table S1: Colony counts & phenotype frequencies of transfected cells

a) DPH1 HRM-PCR (TF eff = 38%)	# of single cells	# of HRM^{+} cells	# of DT resistant cells
	92	6	2

b) DPH1 assay (Int vs KO) (TF eff = 38%)		# of seeded cells	# of DT resistant colonies		# of PM resistant colonies	
20mer SpCas9 scRNA	A	40,000	0	0	17	12
	B		0		13	
	C		0		9	
	D		0		10	
	E	20,000	0	0	11	9
	F		0		7	
	G		0		10	
	H		0		9	
	I	10,000	0	0	3	3
	J		0		2	
	K		0		3	
	L		0		4	
20mer SpCas9 DPH1 gRNA	A	40,000	995	947	29	24
	B		942		12	
	C		899		30	
	D		950		24	
	E	20,000	364	472	18	13
	F		545		13	
	G		543		13	
	H		435		6	
	I	10,000	201	218	7	9
	J		224		10	
	K		219		10	
	L		228		29	

c) DPH2 assay (Int vs KO) (TF eff = 37%*)		# of DT resistant colonies (40,000 seeded cells)		# of PM resistant colonies (40,000 seeded cells)	
20mer scRNA	A	0	0	2	0.5
	B	0		0	
	C	0		0	
	D	0		0	
20 mer SpCas9 DPH2 gRNA	A	144	130	2	1.5
	B	132		2	
	C	122		1	
	D	122		1	

d) DPH1 (Int vs Int+KO) (TF eff = 69*%)		# of PM resistant colonies (40,000 seeded cells)		# of PM+DT resistant colonies (40,000 seeded cells)	
20mer scRNA	A	10	11	0	0
	B	9		0	
	C	10		0	
	D	16		0	
20 mer SpCas9 DPH1 gRNA	A	25	24	15	13
	B	22		13	
	C	25		11	
	D	24		12	

'TF eff.': Transfection efficiency determined by FACS, monitoring fluorescent cells upon transfection with GFP-reporter plasmids (GFP positive cells among all in %). *one assay displayed unusual high GFP positivity and unusual FACS pattern. Average transfection efficacy among all assays was 30 -40%. 'HRM': High resolution melting point PCR positive cells display divergent (biphasic) melting curves compared to wt. 'K.O.' indicates the frequency of cells which carry no functional *DPH* gene and are hence resistant to DT (DT^{r}). 'Int.' indicates the frequency of cells which harbor the *pac* expression cassette i.e. are PM resistant (PM^{r}). 'A-D' are individual results of independent experiments. (a) mono- vs bi-allelic *DPH* inactivation; (b) seeding density correlates with colony number; (c) DT^{r} and PM^{r} upon *DPH2* editing; (d)) DT^{r} and PM^{r} upon *DPH1* editing.

Table S2: Influence of gRNA length on targeted gene inactivation and cassette integration

**DPH1 (gRNA length)** (TF eff = 31%)		**# of DT resistant colonies** (20,000 seeded cells)		**# of PM resistant colonies** (40,000 seeded cells)		**# of DT+PM resistant colonies** (40,000 seeded cells)	
20mer scRNA	A	0	0	7	8	0	0
	B	0		6		0	
	C	0		9		0	
	D	0		10		0	
14 mer SpCas9 DPH1 gRNA	A	0	0	6	8	0	0
	B	0		10		0	
	C	0		8		0	
	D	0		9		0	
16 mer SpCas9 DPH1 gRNA	A	275	284	21	25	7	8
	B	298		26		9	
	C	292		25		10	
	D	271		26		9	
18 mer SpCas9 DPH1 gRNA	A	284	307	19	23	10	10
	B	299		25		12	
	C	321		27		7	
	D	324		22		11	
20 mer SpCas9 DPH1 gRNA	A	417	409	16	15	6	6
	B	406		20		5	
	C	409		16		7	
	D	404		8		7	
22 mer SpCas9 DPH1 gRNA	A	278	284	14	13	6	5
	B	292		12		5	
	C	297		11		4	
	D	267		15		5	
24 mer SpCas9 DPH1 gRNA	A	237	232	13	13	2	3
	B	252		12		4	
	C	228		11		3	
	D	211		15		3	
26 mer SpCas9 DPH1 gRNA	A	161	170	12	11	0	1
	B	155		11		1	
	C	176		12		0	
	D	187		9		1	

'TF eff.': Transfection efficiency was determined by FACS analyses, monitoring frequencies of fluorescent cells upon transfection of MCF7 with GFP-reporter plasmids. Listed are relative numbers of GFP positive cells among all cells in %. Cells which carry no functional *DPH1* gene copy are resistant to DT. Cells which harbor the *pac* expression cassette are hence resistant to PM. 'A-D' indicates individual samples of independent experiments.

Table S3: Phenotype frequencies of MCF-7 transfected with different editing entities

DPH1 editing entities (TF eff = 33%)		# of DT resistant colonies (20,000 seeded cells)		# of PM resistant colonies (40,000 seeded cells)		DT+PM resistant colonies (40,000 seeded cells)	
SpCas9 scRNA	A	0	0	4	4	0	0
	B	0		3		0	
	C	0		5		0	
	D	0		4		0	
SpCas9 DPH1 gRNA	A	476	477	14	11	5	4
	B	492		11		4	
	C	468		9		4	
	D	472		10		3	
SpCas9-HF scRNA	A	0	0	2	1	0	0
	B	0		1		0	
	C	0		0		0	
	D	0		0		0	
SpCas9-HF DPH1 gRNA	A	60	65	9	8	1	0.5
	B	70		10		1	
	C	66		8		0	
	D	62		5		0	
ZFN-DPH1	A	286	283	12	12	4	2.5
	B	292		13		1	
	C	276		10		2	
	D	278		11		3	

MCF-7 cells were transfected with plasmids encoding different genome editing systems (SpCas9, SpCas9-HF, ZFN). The SpCas9 construct was as described before. SpCas9-HF includes the N497A/R661A/Q695A/Q926A substitutions (Kleinstiver *et al.* Nature 529, 490-5, 2016). In parallel, gRNAs were replaced by scRNAs to address non-specific activity. *DPH1*-specific ZFN was obtained from Sigma Aldrich (CompoZr®). The total amount of plasmid DNA (editing entity and donor) for transfection of the initial cell pool of $3x10^6$ cells was as described for the previous experiments. To quantify the transfection efficiency (TF eff=%), GFP-reporter plasmids were transfected aside. GFP-positive cells were counted 24h after transfection by FACS. Defined numbers of cells were seeded and treated with DT, PM, or DT+PM 72 hours thereafter.

Table S4: Phenotypes of MCF-7 exposed to SCR7-pyrazine and/or RS-1 during gene editing

compound	compound addition	mean DT^r colonies #seeded cells: 40.000	mean PM^r colonies #seeded cells: 80.000	% PM^r relative to DT^r colonies
-	4 hrs before transfection	200.5 (213;203;194;192)	8.5 (6;11;8;9)	4.2 %
	18 hrs after transfection	515 (522;513;507;518)	20 (20;24;17;19)	3.9 %
RS-1 (8µM)	4 hrs before transfection	201.5 (196;202;210;198)	14 (11;13;15;17)	**6.9 %***
	18 hrs after transfection	512.5 (512;521;506;511)	15.3 (17;15;14;15)	3.0 %
SCR7 (1µM)	4 hrs before transfection	205.3 (215;193;205;208)	13.3 (11;11;16;15)	**6.5 %***
	18 hrs after transfection	488.8 (486;482;491;496)	25 (26;25;27;22)	5.1 %
RS-1+SCR7 (8µM +1µM)	4 hrs before transfection	175 (183;175;177;165)	14.3 (12;14;15;16)	**8.1 %*****
	18 hrs after transfection	488.3 (492;485;495;481)	10.25 (8;10;6;17)	**2.1 %****

Cells transfected with plasmids for SpCas9-mediated DPH1 editing were seeded in defined numbers (#seeded cells). DT/PM selection started 72hr after transfection. Values (w,x,y,z) indicate colonies obtained in quadruplicate individual experiments. 'SCR7' refers to SCR7-pyrazine (see Methods section). Influence of the time point of the compounds (RS-1, SCR7-pyrazine and RS-1+SCR7-pyrazine) addition. Significant difference of PM resistant relative to DT resistant colonies of treated samples vs. no compound is indicated with *p<0.05, **p<0.01, ***p<0.001.
